# Subluxação da mandíbula para abordagem de bifurcação carotídea alta em paciente com parotidite por contraste iodado: relato de caso e revisão de literatura

**DOI:** 10.1590/1677-5449.001117

**Published:** 2017

**Authors:** Reinaldo Benevides dos Santos, André Brito Queiroz, Ronald José Ribeiro Fidelis, Cicero Fidelis Lopes, José Siqueira de Araújo

**Affiliations:** 1 Hospital Ana Nery – HAN, Departamento de Cirurgia Vascular, Salvador, BA, Brasil.; 2 Hospital Universitário Professor Edgard Santos – HUPES, Departamento de Cirurgia Vascular, Salvador, BA, Brasil.; 3 Universidade Federal da Bahia – UFBA, Faculdade de Medicina, Salvador, BA, Brasil.

**Keywords:** doença das artérias carótidas, parotidite, endarterectomia, angioplastia, fixação maxilomandibular, mandíbula

## Abstract

A doença aterosclerótica das carótidas extracranianas pode resultar em complicações com alta morbidade e mortalidade. A avaliação pré-operatória com exames contrastados de imagem é associada a complicações como a parotidite, além das já bem conhecidas reações alérgicas e da disfunção renal. A bifurcação carotídea alta e a doença aterosclerótica de extensão cranial costumam ser fatores limitantes para o tratamento cirúrgico convencional. Entretanto, quando há contraindicação ao uso de contraste iodado ou impossibilidade do tratamento endovascular, há a necessidade do conhecimento de técnicas cirúrgicas que permitam a realização da endarterectomia com segurança. A subluxação da mandíbula se mostrou uma técnica adjuvante segura e efetiva, de fácil execução e reprodutibilidade, possibilitando o acesso a bifurcações carotídeas altas com boa exposição do campo cirúrgico e permitindo a realização da endarterectomia conforme a técnica padrão. Apresentamos o caso de uma paciente com bifurcação carotídea alta e com limitações para uso do contraste iodado que foi submetida a endarterectomia carotídea após subluxação de mandíbula.

## INTRODUÇÃO

A abordagem da bifurcação carotídea alta ou a presença de extensão distal da doença aterosclerótica demanda tática específica para exposição adequada dos vasos carotídeos, remoção da placa aterosclerótica e arteriorrafia com segurança. A subluxação da mandíbula (SM) tem se mostrado uma técnica efetiva, que evoluiu desde a sua criação.

O objetivo desse artigo é relatar o caso de uma paciente com estenose carotídea sintomática, bifurcação carotídea alta e raro diagnóstico de parotidite pós-contraste, tratada com endarterectomia após SM e revisar a literatura sobre a parotidite por contraste e a técnica de SM. O termo de consentimento livre e esclarecido foi assinado.

## DESCRIÇÃO DO CASO

Paciente do sexo feminino, 55 anos, com episódios recorrentes de hemiparesia esquerda havia três anos. Hipertensa, diabética e dislipidêmica. Alérgica a Sulfametoxazol-trimetropima. Tabagista de 20 maços/ano. Exame neurológico sem alterações.

**Figura 1:**
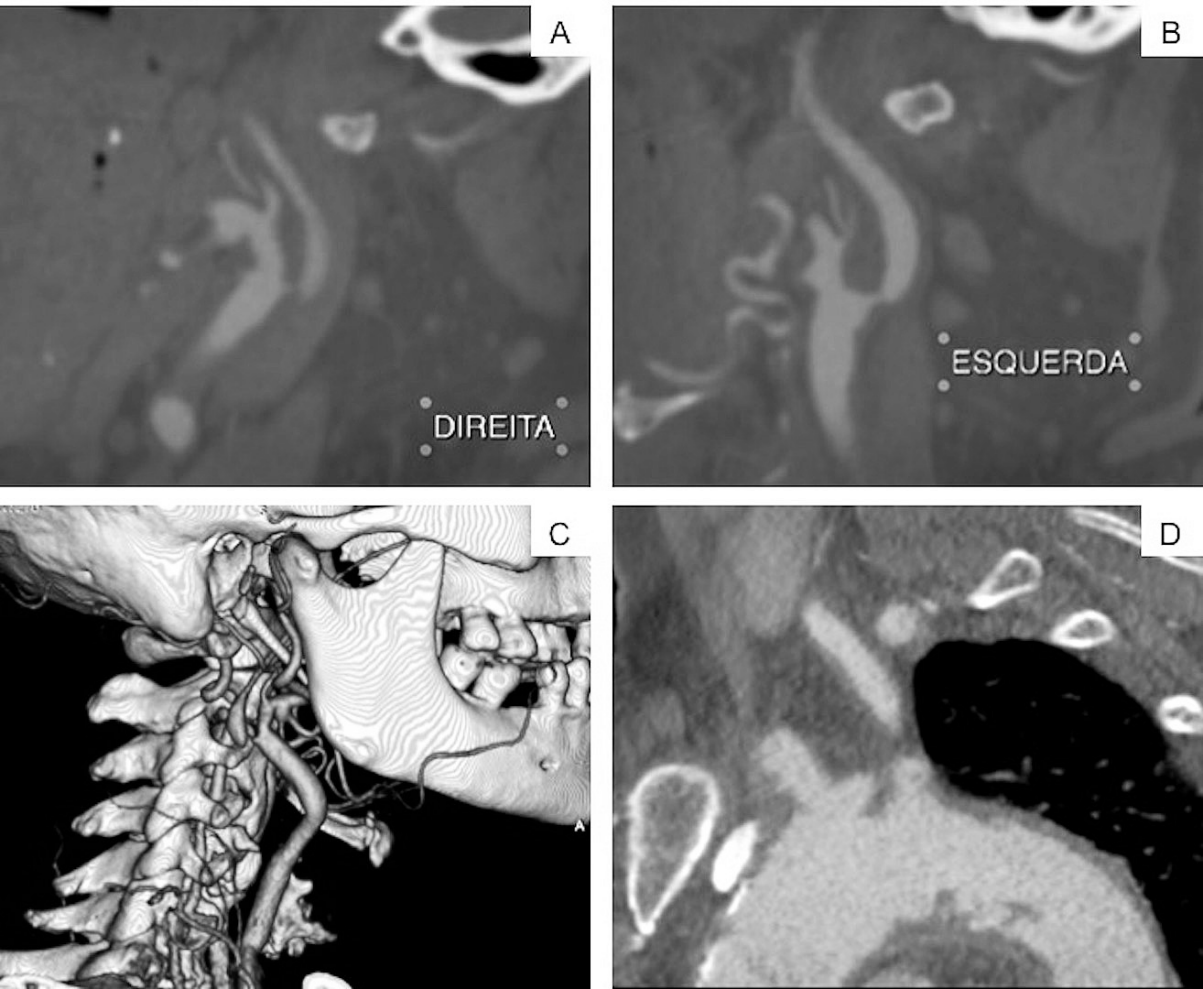
Angiotomografia pré-operatória. (A) Corte sagital de angiotomografia mostrando suboclusão de artéria carótida interna direita (carótida sintomática); (B) Estenose em artéria carótida interna esquerda (carótida assintomática); (C) Imagem de reconstrução mostrando relações anatômicas da bifurcação carotídea direita, retromandibular, com ACI se dirigindo para posterior e com fino calibre até sua porção intracraniana por doença aterosclerótica; (D) Corte sagital de angiotomografia mostrando o arco aórtico repleto de placas.

Tomografia de crânio normal e dúplex descrevendo suboclusão em artéria carótida interna (ACI) direita, estenose maior que 70% em ACI esquerda e artérias vertebrais sem alterações. Ecocardiograma transtorácico com boa função sistólica, câmaras cardíacas de paredes normais e sem trombos intracavitários. Angiotomografia mostrou arco aórtico com placas ateroscleróticas irregulares na sua luz, suboclusão de ACI direita, estenose em torno de 70% da ACI esquerda e vertebrais sem alterações significativas. Chamava atenção a bifurcação carotídea alta, com longa extensão cranial da doença aterosclerótica (Figura 1).

**Figura 2:**
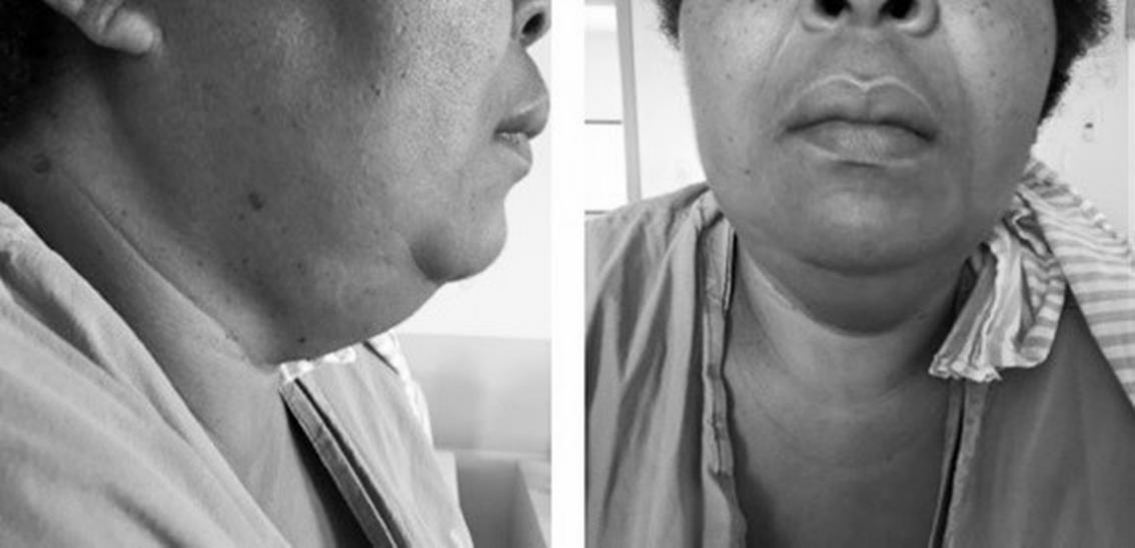
Imagens demonstrando abaulamento cervical. Ultrassonografia confirmou correspondência com aumento das glândulas parótidas. Imagens do quinto dia após exposição ao meio de contraste iodado.

Apresentou abaulamento cervical bilateral, sem sinais inflamatórios ou febre, 48 horas após a angiotomografia. A ultrassonografia mostrou aumento das parótidas, mais pronunciado à direita, com diagnóstico de parotidite pós-contraste. Evoluiu com melhora progressiva após tratamento sintomático (Figura 2). O abaulamento cervical mostrou-se restritivo para endarterectomia. O contraste iodado (único disponível no serviço) para angioplastia poderia resultar em complicações mais graves, então optou-se pela alta hospitalar, com retorno em uma semana para intervenção cirúrgica.

**Figura 3:**
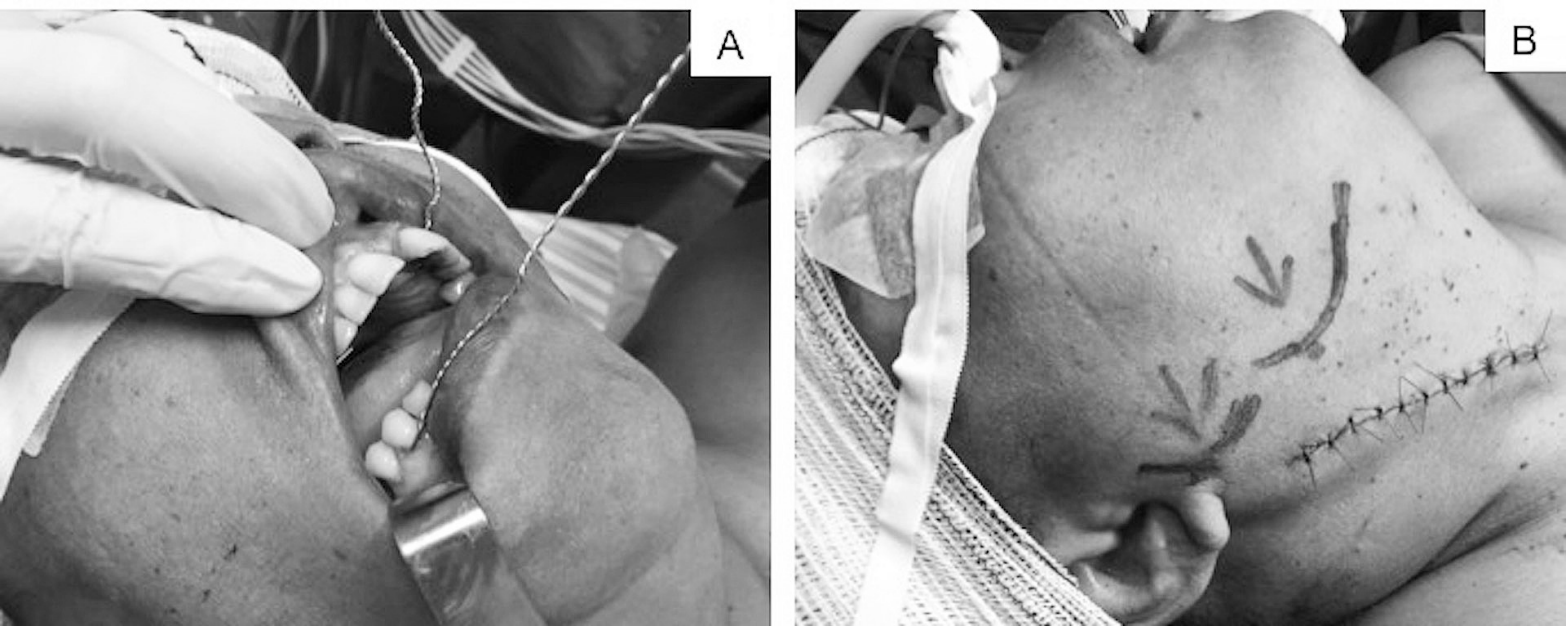
Aplicação da subluxação de mandíbula. (A) Técnica de subluxação de mandíbula em paciente com presença de dentes. Passagem de fio de aço envolvendo unidade dentária da maxila esquerda (canino) e de fio de aço envolvendo unidade dentária da mandíbula em direção ipsilateral ao lado a ser operado. A mandíbula é então subluxada para o lado ipsilateral ao operado através de uma força firme e suave anteromedial e em direção ao lado contralateral. Ao alcançar a posição desejada, os fios são cuidadosamente envolvidos e torcidos em seu próprio eixo; (B) Demonstração do campo cirúrgico ganho através de anatomia topográfica. As marcações correspondem à localização do ângulo da mandíbula. A marcação à esquerda corresponde à localização do ângulo da mandíbula em repouso, e a marcação à direita corresponde à localização do ângulo da mandíbula enquanto a mandíbula estiver subluxada. É notório o ganho no campo cirúrgico, retirando o ângulo da mandíbula da topografia da bifurcação carotídea.

A paciente retornou após 40 dias, com hemiparesia esquerda e disartria. A tomografia de crânio demonstrou AVC isquêmico à direita. Foi submetida a endarterectomia de carótida direita após SM com fixação com fios de aço. O procedimento foi realizado sob anestesia geral e intubação nasotraqueal. Os fios foram fixados nos caninos da mandíbula inferior direita e da maxila superior esquerda e então rodados conjuntamente em torno de seu eixo, mantendo a posição de subluxação (Figura 3).

Seguiu-se com endarterectomia padrão, com secção do ventre posterior do digástrico, reparo do nervo hipoglosso e arteriorrafia com remendo de pericárdio bovino. Não apresentou novo déficit focal nem lesão dentária ou da mucosa oral. Relatou disfagia para sólidos no pós-operatório, que foi resolvida espontaneamente em um mês, atribuída à neuropraxia do hipoglosso por manipulação no intraoperatório. Recebeu alta hospitalar no terceiro dia pós-operatório e, no seguimento de 3 meses, apresentou-se sem eventos neurológicos ou disfunção da articulação temporomandibular (ATM).

## DISCUSSÃO

A endarterectomia carotídea em pacientes sintomáticos ainda é o padrão-ouro, sendo o único método com grau de recomendação I e nível de evidência A para estenoses maiores que 70%^1^.

A altura da bifurcação carotídea é habitualmente descrita na borda superior da cartilagem tireoide, que corresponde ao disco intervertebral C3-C4 (90% dos casos). A bifurcação carotídea é considerada alta se localizada acima do nível de C2 ou com lesões que se estendam além da linha de Blaisdell na avaliação tomográfica. A linha de Blaisdell é traçada do ápice do processo mastoide até o ângulo da mandíbula^2^^-^ 4.

Técnicas prévias com ressecção óssea e muscular ou mobilização da parótida resultavam em maior tempo cirúrgico e mais complicações, frequentemente deformadoras ou incapacitantes. A técnica de SM evoluiu até que uma nova técnica de manutenção da subluxação se mostrou a mais segura, mais simples e menos invasiva^5^^,^ 6.

A SM foi trazida pioneiramente por Fry & Fry^7^, em 1980, para abordagem da ACI distal no trauma, com subluxação bilateral da mandíbula com fixação maxilomandibular, o que tornava o método bastante demorado (90 minutos). Fischer et al., em 1984, citado por Dossa et al.^5^, modificaram a técnica para subluxação unilateral, tornando-a mais rápida (10 minutos) e aplicável a pacientes sem dentes.

A SM demanda anestesia geral e intubação nasotraqueal, permitindo maior mobilidade e segurança no momento da subluxação. De forma firme e cuidadosa, a mandíbula é empurrada anteriormente e inferiormente em direção contralateral ao lado a ser operado (por extensão de 10-15 mm). O côndilo ipsilateral é posicionado no ápice da eminência articular, sem provocar luxação da mandíbula pelo risco de lesão dos ligamentos da ATM, com consequente disfunção da ATM. Na técnica de subluxação, a integridade da cápsula articular temporomandibular é preservada. A mandíbula é mantida subluxada através de fixação na maxila por manobras diversas, com estratégias diferentes dependendo da presença ou não de dentes. É esperado que a subluxação do côndilo mandibular por 10-15 mm propicie a mobilização anterior da mandíbula por 20-30 mm, transformando um campo cirúrgico triangular em retangular^4^^,^ 5.

Pacientes com dentes saudáveis são submetidos a fixação diagonal interdentária com fios de aço. O fio de aço envolve uma ou mais unidades dentárias em posição próxima à raiz, e são rodados conjuntamente em torno de seu eixo. Pacientes com doença periodontal importante ou sem dentes são submetidos a fixação diagonal com auxílio de pinos, parafusos ou pontos passados diretamente nos ossos da mandíbula e maxila, onde se fixam os fios de aço, que são rodados conjuntamente em torno de seu eixo. Outras estratégias são: envolver maxila e mandíbula perialveolar com fios de aço, posicionar parafusos na maxila e mandíbula em direção contralateral ao lado a ser operado ou utilizar próteses dentárias para fixação da subluxação^5^^,^ 6 ^,^ 8.

Em estudo com 1.357 pacientes submetidos a endarterectomia, 43 deles com subluxação, foram comparadas a endarterectomia padrão e a endarterectomia com SM. No grupo subluxação, estavam pacientes com bifurcação carotídea alta ou lesão aterosclerótica além do nível de C2 ou da linha de Blaisdell. Os resultados demonstraram a segurança da técnica de subluxação, pois, comparando mortalidade de causa neurológica, morbidade neurológica perioperatória e lesão nervosa periférica temporária ou permanente, não foram encontradas diferenças nas taxas de complicações, a despeito da dissecção mais extensa nos indivíduos submetidos a SM. O tempo cirúrgico adicional nesse grupo foi, em média, de 15 minutos^4^.

Outros estudos sugerem tratar-se de técnica segura, sem prejuízo à função mastigatória, complicações graves relacionadas ou restrição futura dos movimentos da mandíbula. Podem ocorrer disfunção temporomandibular transitória e sintomas álgicos de curta duração. A lesão nervosa periférica é atribuída mais ao afastamento do que a técnica em si, com resolução em meses. A desvantagem da SM é a necessidade de antecipar sua utilização porque a aplicação no intraoperatório pode ser inviável^5^^,^ 6 ^,^ 8.

Arteriografia, angiotomografia e angiorressonância são exames frequentemente utilizados no planejamento da abordagem carotídea. Os dois primeiros demandam a utilização de contraste iodado, que pode estar relacionado a complicações como anafilaxia, disfunção renal e parotidite.

A parotidite por iodo é uma patologia incomum e pode ocorrer após administração de composto iodado intravenoso, intra-arterial e oral, seja iônico ou não. Não há na literatura descrição da sua incidência. Um estudo japonês que avaliou reações adversas ao contraste iodado, com 337.647 pacientes, não registrou casos de parotidite. Há cerca de 40 casos publicados, sendo os primeiros descritos em 1956 após urografias. Como a fisiopatologia é desconhecida, parece tratar-se de reação idiossincrática. Entretanto, foi descrita indução da parotidite após novas exposições ao contraste, que poderia ser explicada pelo acúmulo tóxico do iodo no sistema ductal das glândulas ou ser resultado da incapacidade do rim em excretar o composto iodado, levando ao seu acúmulo e causando intoxicação da glândula e reação inflamatória^9^^-^ 11.

Zhang et al.^9^ agrupou os casos descritos em língua inglesa e fez uma avaliação descritiva. Identificaram 36 casos: 22 homens e 14 mulheres; idade média de 60 ± 13,6 anos; 19 por injeção endovenosa, 10 por arteriografia, 4 por ingestão oral, 3 por mais de uma via; 31 com apresentação bilateral e 5 com apresentação unilateral; 19 envolvendo a glândula submandibular, 12 envolvendo a glândula submandibular e parótidas, e, ocasionalmente, tireoide e glândulas lacrimais; 9 apresentaram recorrência após nova administração.

Os sintomas se instalam de alguns minutos a até 5 dias após a administração do contraste, e os sintomas persistem por 12 horas a 11 dias. A recorrência é comum se há nova exposição, com descrição de tentativa de dessensibilização com corticosteroides em pacientes com conhecida parotidite por contraste prévia, sem sucesso, sendo aconselhável evitar o seu uso^9^^,^ 12. A complicação mais grave descrita é a paralisia do nervo facial, com necessidade de descompressão. Não há relato de comprometimento de via aérea com risco de morte. Parece tratar-se de patologia autolimitada. O uso de corticoides, anti-inflamatórios e anti-histamínicos não tem benefício comprovado^9^^,^ 13.

O ganho no campo cirúrgico acrescentado pela SM mostrou-se determinante para a execução do procedimento no caso relatado, com boa exposição das estruturas vasculares e nervosas, o que ficou evidente pela ausência de complicações detectadas no pós-operatório.

A parotidite por uso de contraste é patologia rara, pouco conhecida e deve ser lembrada no diagnóstico diferencial. No tratamento da doença carotídea, pode ser um limitante técnico e clínico para o tratamento. A SM mostrou-se técnica segura, reprodutível, de fácil execução e efetiva para o acesso a bifurcações carotídeas altas.
