# Supplementary material for: Subluxação da mandíbula para abordagem de bifurcação carotídea alta em paciente com parotidite por contraste iodado: relato de caso e revisão de literatura
Source: J Vasc Bras. 2017 Oct-Dec;16(4):329–34. [Article in Portuguese] doi: 10.1590/1677-5449.001117 (PMC5944312; doi:10.1590/1677-5449.001117)
Supplement: Translation

# Translation: Subluxation of the mandible for access to high carotid bifurcation in a patient with iodinated contrast induced parotitis: case report and literature review

**Authors:** Reinaldo Benevides dos Santos, André Brito Queiroz, Ronald José Ribeiro Fidelis, Cicero Fidelis Lopes, José Siqueira de Araújo

**Affiliations:** 1 Hospital Ana Nery – HAN, Department of Vascular Surgery, Salvador, BA, Brazil.; 2 Hospital Universitário Professor Edgard Santos – HUPES, Department of Vascular Surgery, Salvador, BA, Brazil.; 3 Universidade Federal da Bahia – UFBA, School of Medicine, Salvador, BA, Brazil.

**Keywords:** carotid artery diseases, parotitis, endarterectomy, angioplasty, jaw fixation techniques, mandible

## Abstract

Atherosclerotic disease of the extracranial carotid arteries can cause complications with high morbidity and mortality rates. The contrast imaging examinations used in preoperative evaluation are associated with complications such as parotitis, in addition to well-known allergic reactions and renal dysfunction. A high carotid bifurcation or atherosclerotic disease that extends distally are often limiting factors for conventional surgical treatment. However, when iodinated contrast is contraindicated or endovascular treatment is not feasible, knowledge of surgical techniques that allow safe endarterectomy is required. Subluxation of the mandible has proven to be a safe and effective adjuvant technique that is easy to perform and reproducible, providing access to high carotid bifurcations with good exposure of the surgical field and allowing endarterectomy to be performed with a standard technique. We present the case of a patient with a high carotid bifurcation and limitations for use of iodinated contrast who underwent carotid endarterectomy after subluxation of the mandible.

## INTRODUCTION

Management of a high carotid bifurcation or atherosclerotic disease with distal extension requires the use of specific tactics to adequately expose the carotid vessels, remove the atherosclerotic plaque and perform arteriorrhaphy with safety. Subluxation of the mandible (SM) has proven an effective technique and has evolved since it was first introduced.

The objectives of this article are to describe the case of a patient with symptomatic carotid stenosis, a high carotid bifurcation, and a rare diagnosis of post-contrast parotitis who was treated with endarterectomy after SM and to review the literature on contrast-induced parotitis and the SM technique. A free and informed consent form was signed.

## CASE DESCRIPTION

The patient was a 55-year-old female with recurrent episodes of left-side hemiparesis with onset 3 years previously. She was hypertensive, diabetic, dyslipidemic and allergic to Sulfamethoxazole-trimethoprim. She was a 20 pack-years smoker. Her neurological examination was normal.

Cranial tomography was normal and the duplex scan showed sub-occlusion of the right internal carotid artery (ICA), stenosis exceeding 70% of the left ICA and vertebral arteries free from abnormalities. Transthoracic echocardiogram showed good systolic function, cardiac chambers with normal walls, and absence of intracavitary thrombi. Angiotomography revealed irregular atherosclerotic plaques in the lumen of the aortic arch, sub-occlusion of the right ICA, stenosis of around 70% of the left ICA and vertebral arteries free from significant changes. Of note was the high carotid bifurcation and the considerable cranial extension of atherosclerotic disease (Figure 1).

**Figure 1:** Preoperative angiotomography. (A) Sagittal angiotomography showing sub-occlusion of the right internal carotid artery (symptomatic carotid); (B) Stenosis of left internal carotid artery (asymptomatic carotid); (C) Reconstruction image showing anatomic relationships between right carotid bifurcation, retromandibular, with ICA coursing posteriorly and with small caliber as far as the intracranial portion due to atherosclerotic disease; (D) Sagittal angiotomography showing the aortic arch with several plaques.

Forty-eight hours after the angiotomography, the patient had developed bilateral cervical swelling, with no signs of inflammation or fever. Ultrasonography showed enlarged parotids, more pronounced on the right, and a diagnosis of post-contrast parotitis was made. She progressively improved after treatment of the symptoms (Figure 2). The cervical swelling proved to be contraindicative for endarterectomy. Since iodinated contrast (the only type available at the service) for angioplasty could cause more serious complications, the decision was taken to discharge her from hospital, to return 1 week later for surgical intervention.

**Figure 2:** Images showing cervical swelling. Ultrasonography confirmed correspondence with enlarged parotid glands. Images from fifth day after exposure to iodinated contrast.

The patient returned 40 days later, with left hemiparesis and dysarthria. Cranial tomography showed right-side ischemic stroke. She was treated with right carotid endarterectomy after SM with fixation using steel wires. The procedure was conducted under general anesthesia with nasotracheal intubation. The wires were attached to the canines of the lower mandible on the right and the upper maxilla on the left and then rotated together around their axis, maintaining the subluxation position (Figure 3).

**Figure 3:** Application of subluxation of the mandible. (A) Technique for subluxation of the mandible of a patient with teeth. A wire is passed around a (canine) tooth of the left maxillary arch and another around a mandibular tooth in a direction ipsilateral to the side to be operated on. The mandible is then subluxated in the ipsilateral direction using a firm and smooth anteromedial force in the contralateral direction. Once the desired position is attained, the wires are carefully twisted around their own axes; (B) Image showing the gain in surgical field with respect to anatomical landmarks. The markings correspond to the locations of the mandibular angle. The left marking corresponds to the position of the mandible at rest and the right marking corresponds to the position of the mandible when it is subluxated. The additional surgical field gained by displacing the angle of the mandible from the topography of the carotid bifurcation is clear.

The operation then proceeded with standard endarterectomy, starting by sectioning the posterior belly of the digastric muscle, with repair of the hypoglossal nerve and arteriorrhaphy with a bovine pericardium patch. No further focal deficit was observed and there were no dental or oral mucosal injuries. She complained of dysphagia for solids during the postoperative period, with spontaneous resolution in 1 month, which was attributed to neuropraxia of the hypoglossal nerve due to intraoperative manipulation. She was discharged from hospital 3 days after the operation and at 3-month follow-up she was free from neurological events or dysfunction of the temporomandibular joint (TMJ).

## DISCUSSION

Carotid endarterectomy remains the gold standard for symptomatic patients and is the only method with grade I recommendation and evidence level A for stenosis exceeding 70%.^1^

The height of the carotid bifurcation is usually described in the superior margin of the thyroid cartilage, which corresponds to the C3-C4 intervertebral disc (in 90% of cases). The carotid bifurcation is considered high if it is located above the level of C2 or has lesions that extend beyond the Blaisdell line on tomography. The Blaisdell line is plotted from the apex of the mastoid process to the angle of the mandible.^2^^-^ 4

Earlier techniques involving resection of bones and muscles or displacement of the parotid resulted in longer surgery times and more complications, frequently deformative or incapacitating. The SM technique evolved until a new technique for maintenance of subluxation proved to be safer, simpler and less invasive.^5^^,^ 6

Subluxation of the mandible was first introduced by Fry and Fry, in 1980,^7^ for access to the distal ICA after trauma, with bilateral subluxation of the mandible and maxillomandibular fixation, which made the method very time consuming (90 minutes). In 1984, Fischer et al., cited by Dossa et al.,^5^ modified the technique to one employing unilateral subluxation, making it quicker (10 minutes) and applicable to patients without teeth.

Subluxation of the mandible requires general anesthesia and nasotracheal intubation, allowing greater mobility and increased safety at the time of subluxation. Firmly and carefully, the mandible is pushed forwards and downwards in the contralateral direction to the side that will be operated (for a distance of 10-15 mm). The ipsilateral condyle is positioned at the apex of the articular eminence, without provoking luxation of the mandible because of the risk of injuring the TMJ ligaments and of consequent TMJ dysfunction. The subluxation technique preserves the integrity of the joint capsule. The mandible is maintained in the subluxated position by fixing it to the maxilla using a variety of methods to achieve different strategies depending on the presence or absence of teeth. It is expected that subluxation of the mandibular condyle by 10-15 mm will enable anterior displacement of the mandible by 20-30 mm, transforming the triangular surgical field into a rectangle.^4^^,^ 5

Patients with healthy teeth are managed with diagonal interdental fixation using steel wires. The steel wires pass round one or more teeth close to the roots, and are twisted together around their axes. Patients with significant periodontal disease or without teeth undergo diagonal fixation using pins, screws or stitches passed directly into the bones of the mandible and maxilla, where the steel wires are attached and twisted together around their axes. Alternative strategies include: wrap steel wires around the maxilla and perialveolar mandible, place screws in the maxilla and mandible in a direction contralateral to the side to be operated or use dental prostheses to fix the subluxation.^5^^,^ 6 ^,^ 8

In a study of 1,357 patients who underwent endarterectomy, 43 of them using subluxation, compared standard endarterectomy and endarterectomy with SM. Patients in the subluxation group had high carotid bifurcation or atherosclerotic lesion above the level of C2 or the Blaisdell line. The results of the study demonstrated the safety of the subluxation technique, since comparisons of mortality from neurological causes, perioperative neurological morbidity and temporary or permanent peripheral nerve damage did not reveal any differences in complication rates, despite the more extensive dissection performed on the SM patients. The mean additional duration of surgery in this group was 15 minutes.^4^

Other studies suggest that this is a safe technique that does not compromise masticatory function, has no serious complications or future restriction of mandibular movement. Transitory temporomandibular dysfunction and pain-related symptoms of short duration can occur. The peripheral nerve damage is more attributable to the displacement than to the technique itself and resolves in months. One disadvantage of SM is the need to predict in advance that it will be used, because it may not be possible to decide to use it intraoperatively.^5^^,^ 6 ^,^ 8

Arteriography, angiotomography, and magnetic resonance angiography are examination commonly employed when planning carotid treatment. The first two of these require iodinated contrast, which can be associated with complications such as anaphylactic shock, renal dysfunction and parotitis.

Iodine-induced parotitis is an uncommon pathology that can occur after intravenous, intra-arterial or oral administration of iodine compounds, whether ionic or not. There are no descriptions of its incidence in the literature. A Japanese study that evaluated adverse reactions to iodinated contrast, with 337,647 patients, did not record any cases of parotitis. There are around 40 cases published, the first of which was described in 1956 and occurred after urography. Since pathophysiology is unknown, it appears to be an idiosyncratic reaction. However, induction of parotitis has been described after repeated exposure to contrast, which could be because of a toxic accumulation of iodine in the ductal system of the glands or could be the result of an incapacity of the kidneys to excrete the iodine compound, leading to accumulation and causing intoxication of the gland and inflammatory reaction.^9^^-^ 11

Zhang et al.^9^ collected cases described in English and performed a descriptive analysis. They identified 36 cases: 22 men and 14 women; with mean age of 60 ± 13.6 years; 19 administered by intravenous injection, 10 during arteriography, 4 by oral route, 3 by more than one route; 31 with bilateral presentation and 5 with unilateral presentation; 19 involving the submandibular gland, 12 involving the submandibular and parotid glands, and, occasionally, the thyroid and lacrimal glands; and 9 cases that exhibited recurrence after repeated administration.

Symptoms emerged from a few minutes to up to 5 days after administration of the contrast and lasted for 12 hours to 11 days. Recurrence is common if exposure occurs repeatedly and there are descriptions of attempts at desensitization with corticosteroids in patients with known prior contrast-induced parotitis, which were unsuccessful, showing that it is advisable to avoid administration.^9^^,^ 12 The most severe complication described was paralysis of the facial nerve, requiring decompression. There were no reports of airway involvement with risk of death. The pathology appears to be self-limiting. There is no proven benefit to administration of corticosteroids, anti-inflammatories, or antihistamines.^9^^,^ 13

The expansion of the surgical field afforded by SM proved to be key to enabling execution of the procedure in the case described, offering good exposure of vascular and nerve structures, exemplified by the absence of complications during the postoperative period.

Parotitis due to use of contrast is a rare pathology that is little known and should be considered in differential diagnosis. It can impose technical and clinical limitations in treatment of carotid disease. Subluxation of the mandible proved to be a safe and reproducible technique that was easy to perform and effective for accessing high carotid bifurcation.
